# The association of visual memory with hippocampal volume

**DOI:** 10.1371/journal.pone.0187851

**Published:** 2017-11-08

**Authors:** Andrea R. Zammit, Ali Ezzati, Mindy J. Katz, Molly E. Zimmerman, Michael L. Lipton, Martin J. Sliwinski, Richard B. Lipton

**Affiliations:** 1 Saul B. Korey, Department of Neurology, Albert Einstein College of Medicine, Bronx, NY, United States of America; 2 Einstein Aging Study, Albert Einstein College of Medicine, Bronx, NY, United States of America; 3 Department of Neurology, Montefiore Medical Center, Bronx, NY, United States of America; 4 Department of Psychology, Fordham University, Bronx, NY, United States of America; 5 The Gruss Magnetic Resonance Research Center and Departments of Radiology, Psychiatry and Behavioral Sciences and the Dominick P. Purpura Department of Neuroscience, Albert Einstein College of Medicine, Bronx, NY, United States of America; 6 Department of Radiology, Montefiore Medical Center, Bronx, NY, United States of America; 7 Department of Human Development and Family Studies, Pennsylvania State University, University Park, Pennsylvania, United States of America; 8 Department of Epidemiology and Population Health, Albert Einstein College of Medicine, Yeshiva University, Bronx, NY, United States of America; Nathan S Kline Institute, UNITED STATES

## Abstract

**Background:**

In this study we investigated the role of hippocampal volume (HV) in visual memory.

**Methods:**

Participants were a subsample of older adults (> = 70 years) from the Einstein Aging Study. Visual performance was measured using the Complex Figure (CF) copy and delayed recall tasks from the Repeatable Battery for the Assessment of Neuropsychological Status. Linear regressions were fitted to study associations between HV and visual tasks.

**Results:**

Participants’ (n = 113, mean age = 78.9 years) average scores on the CF copy and delayed recall were 17.4 and 11.6, respectively. CF delayed recall was associated with total (β = .031, *p* = 0.001) and left (β = 0.031, *p* = 0.001) and right HVs (β = 0.24, *p* = 0.012). CF delayed recall remained significantly associated with left HV even after we also included right HV (β = 0.27, *p* = 0.025) and the CF copy task (β = 0.30, *p* = 0.009) in the model. CF copy did not show any significant associations with HV.

**Conclusion:**

Our results suggest that left HV contributes in retrieval of visual memory in older adults.

## Introduction

Medial temporal lobe structures, in particular the hippocampus, play an undisputed role in memory processes. Prior studies, investigating the role of hippocampus in memory processes, have suggested differential areas of brain activation for verbal and spatial ability, with right hippocampal volume (HV) more strongly associated with spatial memory, while left HV more storongly associated with verbal memory [1–3][4][5][6]. Structural MRI studies in Alzheimer’s disease (AD) research have also shown these associations [7, 8]. However, few studies have focused on the roles of visual memory in the hippocampus. There has been increasing evidence showing that measures of visual memory are key markers of hippocampal integrity [9], have better diagnostic value, and can discriminate individuals at various stages on the diagnostic scale (i.e. MCI, AD) [10–12].

The Complex Figure (CF) subtest from the Repeatable Battery for the Assessment of Neuropsychological Status (RBANS) incorporates a wide range of cognitive abilities, including visual perception, constructional praxis, planning and organization, and visual memory [13]. It differs from tests of spatial memory in that it uses a figural reproduction paradigm [14]. The delayed aspect of it is thought to reflect organizational and visual perceptual memory [13]. Clinically, this task has lately offered insights into research on mild cognitive impairment (MCI) and AD [15]. Studies on AD subjects have shown that visual and spatial deficits appear before semantic and verbal episodic memory impairment [16, 17]. Studies have also specifically shown that visual, rather than verbal measures, predict incident AD up to ten years prior to a diagnosis [10]. One study even reported that of eight neuropsychological tests, a visual memory measure, the Benton Visual Retention Test, was the most sensitive in detecting dementia [18]; this visual memory test demonstrated accelerated decline 6 years before AD onset [19]. Even in pathology studies, more reported errors on visual memory tasks in older adults have been associated with cerebral amyloid deposition, a marker of AD, 20 years prioir to autopsy [20]. Some studies have even attempted to link visual memory tests in MCI and AD patients with hippocampal laterality [21]; however, research in this area is still limited. Identifying tests that may be sensitive to change in hippocampal volume may offer a promising approach to diagnose AD at a presymptomatic stage. Therefore, investigation on how visual measures relate to hippocampal volume is warranted. Furthermore, understanding of the relationship between episodic memory measures and hippocampal volume might be crucial in studies using these measures for early detection of AD.

In this study, our aim was to investigate the association between total and unilateral hippocampal volume with visual ability using the CF subtests. As a secondary aim we examined the associations between performance on CF subtests and cognitive domains of general fluid ability, processing speed, and episodic memory to find out if these visual tasks are related to different latent constructs within the structure of intelligence. Since within the structure of intelligence, visual and verbal memory share unique variance with episodic memory, while spatial memory shares variance with general fluid ability [22], we hypothesized strong associations between visual memory (the delayed aspect of CF) and left HV.

## Methods

### Sample characteristics

This was a cross-sectional study conducted on 113 older adults from the Einstein Aging Study (EAS) who participated in MRI studies between July 2011 and October 2014. The EAS study design and methods have been described previously [23]. Briefly, the EAS is an ongoing community-based volunteer sample of individuals over the age of 70 living in the Bronx, New York. Participants are systematically recruited from Bronx County voter registration lists from the New York City Board of Elections. Participants were excluded from this sub-study if they had visual and/or auditory impairment that interfered with neuropsychological testing, psychiatric symptomatology that interfered with test completion, were non-English speakers, were nonambulatory, had a dementia diagnosis or were ineligible for an MRI (e.g. due to metallic implants, claustrophobia, etc.). This study is a sub-study of the parent Einstein Aging Study Program Project. As part of this PPG, each participant is administered a mental status exam to determine if they have capacity to consent. This procedure is done annually and reviewed by a licensed neuropsychologist or physician before they undergo the Clinical Core battery. In addition, before they are invited to participate in any sub-studies such as the present one, they must demonstrate appropriate mental capacity. Written informed consent for this sub-study was obtained for neuropsychological assessment at the baseline clinical visit and consent for MRI was obtained on the day of the procedure. The study protocol was approved by the Albert Einstein College of Medicine Institutional Review Board.

### Cognitive tests included in the principal components analysis (PCA)

We used 9 cognitive tests to generate a PCA on the 113 participants’ scores to form robust cognitive composites of general fluid ability, processing speed, and episodic memory from selected subtests (Digit Symbol Coding, Block Design, and Digit Span) from the Wechsler Adult Intelligence Scales 3^rd^ edition [24]; Logical Memory from the Wechsler Memory Scales 3^rd^ edition [25]; Categories (Animals, Fruits and Vegetables) [13]; Trail Making Tests A and B [26]; Free and Cued Selective Reminding Test [27] and the Stroop Golden Color Word Score [28].

### Visual memory

Visual memory was assessed using the Complex Figure copy and Complex Figure delayed recall subtests from the Repeatable Battery for the assessment of Neuropsychological Status (RBANS) [29]. This test measures immediate visual ability and delayed visual episodic memory and is language-free. The first part of this test involves the immediate free-hand copying of a very detailed line drawing. In the second part, which is the delayed part, the participant is required to recall and reproduce the figure from memory after a 20-minute delay. Scores are based on accuracy of drawing and placement. Possible scores range from 0 to 20. This test was administered on the same day that the cognitive tests included in the PCA were administered.

### MRI acquisition/MRI analysis

Imaging was performed using a 3.0 T MRI scanner (Achieva Quasar TX; Philips Medical Systems, Best, the Netherlands) with a 32-channel head coil (Sense Head Coil; Philips Medical Systems, Best, the Netherlands). T1-weighted whole-head structural imaging was performed using sagittal three-dimensional magnetization-prepared rapid acquisition gradient echo (MP-RAGE) with TR/TE 9.9/4.6ms; 240 mm^2^ FOV; 240×240 matrix; partition thickness, 1 mm; and parallel acceleration factor 2.0.

MRI data was processed using the FreeSurfer software package (version 5.2, available at http://surfer.nmr.mgh.harvard.edu/). Image processing methods in the EAS have been previously described in detail [30]. Total HV was segmented using FreeSurfer’s standard segmentation procedure using a probabilistic brain atlas [31]. Additionally, for each subject, the estimated total intracranial volume (TICV) was calculated within FreeSurfer by the procedure described by Buckner et al. [32]. Visits between cognitive testing and MRI were approximately two weeks apart.

### Statistical analysis

Statistical analyses were conducted using IBM SPSS Statistics for Windows, Version 22.0 (Armonk, NY: IBM Corp).

We used principal components analysis (PCA) by varimax rotation to generate cognitive components from the nine neurocognitive tests listed above (results in S1 Table). The associations between the standardized coefficients from the latent PCA components and the total score from the Complex Figure copy and delayed recall tasks were examined using multivariate linear regression adjusting for age, sex, and years of education. A Sidak correction [33] factor with an adjusted *p*-value of 0.0167 for the cognitive components (α = 0.05, three cognitive components) was used to correct for Type I error.

We then ran a series of multivariate linear regression analyses to determine if Complex Figure is associated with total, left, and right hippocampal volume. In the first set of models we investigated the effect of total HV on visual memory; we then analyzed the effect of right HV and left HV separately. In all models we adjusted for age, sex, race, years of formal education, and total intracranial volume (TICV). In analyzing the laterality of the hippocampi, we added a second model, that included both HVs in the same model. In analyzing CF recall, we further adjusted for the scores of the CF copy to control for the effects of the copy part in the recall task. Since only a limited number of associations were run and we wanted to avoid overadjusting we did not correct for Type 1 error here.

## Results

The mean age of the sample was 78.9 years and 39.8% were male. Global cognitive function using the Blessed Information Memory Concentration [34] was 2.1 (SD = 2.4). Mean scores on measures of visual performance and memory using CF copy and delayed recall were 17.4 and 11.6 (SDs = 2.0 and 3.8). Table 1 summarizes the sample characteristics for the 113 participants.

**Table 1:** Characteristics of the sample and correlation measures with visual measures and hippocampal volume.

	Total sample (n = 113)	RBANS CF copy (r/t^1^)	RBANS CF delayed recall (r/t)	Left HV (r/t)	Right HV (r/t)	Total HV (r/t)
Age, years (SD)	78.9 (5.1)	-0.06	-0.23**	-0.35**	-0.51**	-0.45**
Males (%)	45 (39.8)	0.83	1.45	2.66**	2.56*	2.80**
Education, years (SD)	14.4 (3.4)	0.23**	0.19*	-0.04	-0.02	-0.03
African American (%)	51 (38.3%)	0.03	-0.50	-0.26	-0.03	-0.15
BIMC	2.1 (2.4)	-0.09	-0.28**	-0.25*	-0.12	-0.18*
RBANS CF copy (SD)	17.4 (2.0)	-	0.34**	-0.02	0.02	0.00
RBANS CF delayed recall (SD)	11.6 (3.8)	0.34**	-	0.32**	0.24**	0.30**
Total hippocampal volume, cm^3^ (SD)	6.5 (8.1)	0.00	0.30**	0.93**	0.92**	-
Right Hippocampal Volume, cm^3^ (SD)	3.2 (0.4)	0.02	0.24**	0.72**	-	0.92**
Left Hippocampal Volume, cm^3^ (SD)	3.2 (0.4)	-0.02	0.32*	-	0.72**	0.93**
Total intracranial volume, cm^3^, (SD)	1358 (213)	0.15	0.17	0.18*	0.15	0.18*

*Note*. BIMC = Blessed Information Memory Concentration. RBANS = Repeatability Battery for the Assessment of Neuropsychological Status. CF = Complex Figure.

*p < .05

**p < .01

^1^ We used the Spearman correlation (r) for continuous variables, and the t-test (t) for categorical variables.

S1 Table shows the cognitive tests that made up the cognitive constructs of episodic memory, processing speed, and general fluid ability in our sample.

In Table 2 the associations between the three cognitive constructs and the CF tasks are presented. After adjustments for demographic covariates and corrections for multiple testing, results showed significant associations between CF copy and g_f_ (β = 0.30, *p* = 0.002), and CF delayed recall and episodic memory (β = 0.43, *p*≤0.001).

**Table 2:** Linear regressions between cognitive components and Complex Figure–copy and delayed recall tasks.

	Complex Figure–copy			Complex Figure–delayed recall		
	β	t	p	β	t	p
Processing speed	0.02	0.20	.840	0.19	2.20	.030
Episodic memory	0.14	1.53	.128	**0.43**	**5.22**	**≤.001**
g_f_	**0.35**	**3.51**	**.001**	0.22	2.27	.025

Adjusted for age, sex, race, and education. g_f_ = general fluid ability. β = standardized regression coefficient. Corrected for multiple testing (*p* < .0167 was considered statistically significant). Bold indicates significant associations.

Table 3 shows results from linear regression analyses with the CF copy and CF delayed recall tasks as the dependent variables and total and left and right hippocampal volume as independent variables, while adjusting for age, sex, race, education, and total intracranial volume. We found a significant association between larger left and right HVs and better performance on the CF delayed recall task (left HV: β = 0.31, *p* = 0.001; right HV: β = 0.24, *p* = 0.012). After including both HVs in the same model, the left HV remained significant with CF delayed recall (β = 0.27, *p* = .025). This association remained significant after further adjustment for the copy part of CF (β = 0.30, *p* = 0.009). There were no associations between HV and the CF copy test. We also found a positive association between total HV and performance on the CF delayed recall task (β = 0.31, *p* = 0.001). No associations were found with the CF copy task.

**Table 3:** Linear regression for the effect of left, right, and total hippocampal volume on Complex Figure copy and delayed recall tasks.

Outcome	Test used	Model	Predictor	β	t	*p*	*r*^*2*^
Visual copy	CF–copy	Model 1	Left HV	-0.07	-0.67	.503	0.045
			Right HV	0.01	0.01	.989	0.042
		Model 2	Left HV	-0.11	-0.89	.376	0.048
			Right HV	0.08	0.69	.560	0.048
Visual memory	CF–delayed recall	Model 1	**Left HV**	**0.31**	**3.40**	**.001**	**0.173**
			**Right HV**	**0.24**	**2.54**	**.012**	**0.141**
		Model 2	**Left HV**	**0.27**	**2.26**	**.026**	**0.175**
			Right HV	0.06	0.52	.608	0.175
		Model 3	**Left HV**	**0.30**	**2.66**	**.009**	**0.255**
			Right HV	0.04	0.35	.729	0.255
Visual copy	CF–copy	Model 1	Total HV	-0.04	-0.35	.724	0.048
Visual memory	**CF–delayed recall**	Model 1	**Total HV**	**0.31**	**3.29**	**.001**	**0.168**
		Model 2				**NA**
		Model 3	**Total HV**	**0.32**	**3.55**	**0.001**	**0.255**

Note. β = standardized regression coefficient. CF = Complex Figure. Model 1: Adjusted for age, sex, education, race, and total intracranial volume. Model 2: Adjusted for Model 1 + Left and Right HV simultaneously. Model 3: Adjusted for Model 1 + Model 2 + Complex Figure–Copy. Bold indicates significant associations.

Upon testing for model fit (Table 3) we found that in CF copy, the left HV had a better fit (r^2^ = 0.045) than the right HV (r^2^ = 0.042). For CF delyed, the left HV also had a better fit (r^2^ = 0.173) than the right (r^2^ = 0.141). When both left and right HVs were entered the model together the fit improved (r^2^ = 0.175), and when CF copy was also included in the model the fit improved further to 0.255. The fit for CF delayed recall was also better than for CF copy when assessing total HV (r^2^ = 0.048 for CF–copy vs r^2^ = 0.168 for CF–delayed). With CF copy as an adjustment in the CF delayed recall the fits improveed further to 0.255.

## Discussion

In this study we explored visual performance in copy and recall tasks in non-demented older adults using the RBANS Complex Figure copy and delayed recall tasks. We showed that delayed visual memory as measured by RBANS Complex Figure delayed recall is associated with left hippocampal volume.

Results from our cognitive component scores on the PCA showed an association between the immediate component of the visual test (CF copy) and g_f_; and an association between the delayed component of the visual test (CF delayed recall) and episodic memory. The association between the copy task and g_f_ illustrates the purely visual aspect of the CF task, where an understanding of line orientation and an understanding of the relations between patterns and spaces are needed to complete the task. In addition, the strong association between episodic memory and the delayed recall task supports previous studies that have shown shared variance between visual and verbal episodic memory [22].

As suggested by many other studies [35–37], hippocampal volume in healthy older adults is associated with cross-sectional performance on episodic memory tests. Furthermore, hippocampal atrophy has been found to predict the rate of cognitive decline based on tests of verbal episodic memory [35, 38]. Our group has previously shown a strong association between left hippocampal volume and verbal memory in the same population [36]. Our group and other studies have also indicated that right hippocampus plays a critical role in spatial memory and navigational abilities [37, 39]. In this study we showed that bilateral HV is associated with visual memory performance, with left HV showing stronger associations.

Studies on the relationship between hippocampal function and visual memory in older adults are limited, and published reports of this association in other populations have been less consistent [14, 40, 41]. Some studies have found visual memory to be associated with bilateral HV [21, 41, 42], which was supported in our study. In a series of volumetric MRI studies in London taxi drivers, performance on visual memory measured by performance on the Complex Figure test was directly associated with total HV [41]. In one study on patients with both right and left temporal epilepsy, positive correlations were only found between left hippocampal volume and Complex Figure delayed recall scores [14]. And in a study on patients with multiple sclerosis, an association was reported between tests of visual memory and the left hippocampus [43]. Lastly, in other studies of patients with temporal lobe epilepsy, a combination of visual memory tests failed to show sensitivity or specificity to lateralize the hippocampus [40, 44]. In the current study, better performance in visual memory as measured by the CF delayed recall task was associated with a larger left HV. This result suggests that left hippocampus contributes in retrieval of visual memory in older adults.

In future research, the development of additional nonverbal memory constructs is encouraged to further advance our understanding of how these abilities map onto the brain and its functions across age-groups and patient populations, particulary those with amnestic mild cognitive impairment (aMCI) and AD. This is especially because visual memory measures have been found to predict future onset of dementia [10, 18–20]; however studies have been lacking in associating these measures with structural brain imaging. If visual memory measures are able to identify subjects in the preclinical phase of AD, we would be in a better situation to identify individuals at risk earlier.

A strength of this study is the specific age group of participants which helps in drawing conclusions in older adults. Although previous research [45] has shown consistencies across different age groups it is important to replicate these studies in other populations. One limitation is that we only have a single indicator variable to represent visual memory. Furthermore, the scoring criteria of Complex Figure accuracy are subjective in nature, and may contribute to the results. Future research should incorporate other tests of visual memory to provide a more complete representation of visual memory abilities. Lastly, the hippocampus is not a stand-alone structure; it is embedded within the medial temporal lobes and surrounded with neural networks, which activities and functions are yet to be known. Future larger imaging studies are needed to further investigate the role of visual memory and its relations to verbal and spatial memory in the brain.

In conclusion, we showed that better episodic memory performance, and larger left and right HV were associated with higher visual memory performance in non-demented older adults. Further studies are required to explore the nature of the visual memory system and its implication in MCI and AD populations.

## Supporting information

S1 Table Principal components analysis of nine cognitive tests. (DOCX)
