# Supplementary material for: The association of visual memory with hippocampal volume
Source: PLoS One. 2017 Nov 8;12(11):e0187851. doi: 10.1371/journal.pone.0187851 (PMC5678713; doi:10.1371/journal.pone.0187851)
Supplement: S1 Table — (DOCX) [file pone.0187851.s001.docx]

**Supporting Information**

S1 Table. Principal components analysis of nine cognitive tests.

| Variable | Processing Speed | Episodic Memory | General fluid ability |
| --- | --- | --- | --- |
| % of variance | 40.8 | 8.3 | 5.7 |
| Cognitive tests |  |  |  |
| Trail Making Test A | **-0.76** | -0.15 | -0.22 |
| Trail Making Test B | **-0.68** | -0.30 | -0.36 |
| Digit Symbol Coding | **0.59** | 0.32 | 0.33 |
| FCSRT Free Recall | 0.38 | **0.73** | -0.17 |
| CAT | 0.37 | **0.60** | 0.18 |
| Logical Memory | 0.01 | **0.61** | 0.40 |
| Block Design | 0.24 | 0.15 | **0.67** |
| Digit Span, total | 0.16 | 0.01 | **0.46** |
| Stroop Golden Color Word Score | 0.39 | 0.40 | **0.46** |

*Note.* Bold shows loading coefficients above .45. FCSRT = Free and Cued Selective Reminding Test. CAT = Categories.
